# Plitidepsin in combination with dexamethasone (ADMYRE trial) *versus* an external control arm of pomalidomide plus dexamethasone in patients with relapsed/refractory multiple myeloma

**DOI:** 10.1007/s00277-026-06811-w

**Published:** 2026-01-17

**Authors:** Heinz Ludwig, Evangelos Terpos, Mario Boccadoro, Sara Martínez, Carmen Kahatt, Javier Jiménez, Antonio Nieto, Sonia Extremera, Javier Gómez, Vicente Alfaro, Ruthanna Davi, Xiang Yin, María Victoria Mateos

**Affiliations:** 1 Department of Medicine, Center for Oncology, Hematology and Palliative Care, Wilhelminen Cancer Research Institute, Wilhelminen Hospital, Vienna, Austria; 2 Department of Clinical Therapeutics, School of Medicine, National and Kapodistrian University of Athens, Athens, Greece; 3 European Myeloma Network, EMN, Torino, Italy; 4 Department of Molecular Biotechnology and Health Sciences, University of Torino, Torino, Italy; 5 Clinical R&D, Pharma Mar, Colmenar Viejo, Madrid Spain; 6 Medidata AI , Medidata Solutions, a Dassault Systèmes Company, New York, U.S.; 7 Departamento de Hematología, Hospital Universitario de Salamanca. Paseo de San Vicente, Salamanca, 58-182. 37007 Spain

**Keywords:** Plitidepsin, Pomalidomide, Low-dose dexamethasone, External control arm

## Abstract

Plitidepsin (P) is a marine-derived anticancer compound isolated from the tunicate *Aplidium albicans*. P plus low-dose dexamethasone (LD-DXM) was evaluated *versus* LD-DXM alone in patients with relapsed/refractory multiple myeloma (r/r MM) in the randomized phase III ADMYRE trial. In absence of a randomized study with P + LD-DXM vs. POM + LD-DXM, a direct matched comparison between P + LD-DXM (ADMYRE data) and pomalidomide (POM) + LD-DXM as an External Control Arm (ECA) was conducted using individual patient-level data from several contemporary POM + LD-DXM trials with a similar design. A first analysis (ECA1) showed that P + LD-DXM was non-inferior to POM + LD-DXM in terms of median overall survival (OS): 11.8 vs. 13.9 months; HR = 1.009 (95%CI, 0.812–1.254; *p* = 0.9336). Safety profile showed a lower rate of grade ≥ 3 hematological treatment-related adverse events (TRAEs) (neutropenia 2.5% vs. 37.1%; thrombocytopenia 2.5% vs. 13.2%) and infections (8.1% vs. 18.7%) for P + LD-DXM, and a higher rate of grade ≥ 1 gastrointestinal TRAEs (52.8% vs. 27.4%), grade ≥ 3 blood creatine phosphokinase (14.3% v*s.* 0%) and grade ≥ 3 myalgia (5.6% vs. 0%). A second analysis (ECA2) compared POM + LD-DXM with the LD-DXM alone arm included in ADMYRE and showed a treatment effect in OS (HR = 0.762; 95%CI, 0.566–1.026) similar to that observed in ADMYRE (HR = 0.797, 95%CI, 0.596–1.067). Safety profile of POM + LD-DXM was associated to a higher rate of TRAEs, as expected for a combination. In conclusion, P + LD-DXM can be an alternative therapeutic option in r/r MM as this comparison shows that P + LD-DXM is non-inferior in OS to POM + LD-DXM with an advantageous safety profile in terms of hematological and infection events.

**Supplementary Information:**

The online version contains supplementary material available at 10.1007/s00277-026-06811-w.

## Introduction

Plitidepsin is a cyclic depsipeptide originally extracted from the tunicate *Aplidium albicans* and currently produced by chemical synthesis that has potent in vitro activity against primary multiple myeloma (MM) tumor cells and a broad spectrum of human MM cell lines [1]. Plitidepsin interacts with the eukaryotic elongation factor eEF1A2, an oncogene overexpressed in MM cells [2]. This interaction leads to early oxidative stress, Rac1 activation, initiation of the MAPK cascade and cyclin down-regulation, induction of endoplasmic reticulum stress, and activation/deactivation of several cytoplasmic factors. These events finally result in induction of apoptosis, perturbation of the cell cycle, and a potent antiproliferative effect.

Plitidepsin (P) 5 mg/m^2^ i.v. on Day 1 and 15 every four weeks (q4wk) plus low-dose dexamethasone (LD-DXM) (40 mg orally on Day 1, 8, 15 and 22 q4wk) was evaluated *versus* LD-DXM alone in patients with relapsed/refractory multiple myeloma (r/r MM) exposed to ≥3–6 prior treatment lines in the randomized phase III ADMYRE trial [3]. Low-dose dexamethasone regimen was used in combination with plitidepsin as experimental arm and as single agent in the control arm of ADMYRE trial because, based on data from last available publications [4] at the time of protocol writing, dexamethasone administered in combination at reduced doses was associated with a better safety profile while reporting better survival. This was agreed with the Health Authorities. ADMYRE showed a statistically significant prolongation of progression-free survival (PFS) with P + LD-DXM and a tolerable safety profile [3]. Based on these findings, the P + LD-DXM combination was approved in Australia in 2018 for the treatment of patients with r/r MM.

External Control arms (ECAs) help to evaluate the effectiveness of a new treatment by comparing its outcomes with those of a similar group of patients who received standard care or a different treatment. An ECA can be created from patient-level data from individuals who were external to the investigational trial and selected with statistical methods such as propensity score modeling to provide confidence that the baseline characteristics of the selected external patients are balanced and comparable with the baseline characteristics of the patients treated with the investigational product to whom they are compared [5, 6]. After an ECA is created with a similar distribution of baseline characteristics to the investigational arm patients, the differences in outcomes between the two groups of patients can be reliably attributed to the investigational product.

Historical clinical trials (HCT) data and real-world (RWD) data are the common data sources to form an ECA, although HCT data has advantages over RWD data for ECA construction including availability of traditional clinical trial endpoints and covariates, arising from a more similar patient pool in that these patients were participating in a clinical trial, and a rigorous and more complete collection of data often done at prestigious medical centers [7–9]. A scoping review examining the use of ECAs in oncology from 1996 to 2022 showed that 44% of them were conducted in blood-related cancer studies [10]. ECAs based on HCT or RWD data have been previously used to conduct adjusted comparisons of treatments in r/r MM: teclistamab *versus* an ECA of four daratumumab clinical trials (POLLUX, CASTOR, EQUULEUS, and APOLLO) [11]; ciltacabtagene autoleucel in CARTITUDE-1 trial *versus* an ECA of three daratumumab clinical trials (POLLUX, CASTOR, and EQUULEUS) [12]; talquetamab in MonumenTAL-1 trial *versus* an ECA of two observational RWD studies (LocoMMotion and MoMMent) [13]; or talquetamab in MonumenTAL-1 trial *versus* an ECA from patients in the Flatiron Health database [14].

Pomalidomide (POM) is an appropriate comparator to plitidepsin as it was approved while ADMYRE trial was being conducted and both drugs were evaluated in a similar population: patients with r/r MM, who receive at least two prior treatment regimens that include both a proteasome inhibitor and an immunomodulatory drug. POM plus LD- DXM was evaluated in the pivotal randomized phase III study MM-003 in r/r MM patients [15]. In an indirect comparison (ADMYRE *versus* MM-003), the median overall survival of POM + LD-DXM (OS = 12.7 months) was quite similar to the median OS of 11.6 months observed for P + LD-DXM in ADMYRE [3], while the safety profile of P + LD-DXM compared favorably with respect to POM + LD-DXM. Grade ≥ 3 neutropenia (16% for P + LD-DXM vs. 48% with POM + LD-DXM) and febrile neutropenia (0.6% vs. 10%) were lower with P + LD-DXM. Severe complications derived from low neutrophil counts were less frequent with P + LD-DXM: grade 2 infection (1.8% vs. 30% with grade ≥ 3 infection with POM + LD-DXM); grade ≥ 3 pneumonia (2.6% vs. 14%). Gastrointestinal and muscular events were more common with P + LD-DXM.

To provide a direct matched comparison with an ECA between P + LD-DXM (ADMYRE data) and POM + LD-DXM, we show here an analysis using individual patient-level data from several contemporary POM trials.

## Materials and methods

An ECA was prospectively designed using patient-level external data from HCT including the population with the same disease condition evaluated in ADMYRE (adult patients with r/r MM). Only patients from HCT who were assigned to receive POM + LD-DXM were eligible for inclusion in the ECA, which was constructed using a propensity score approach to balance the ECA patients with the important baseline characteristics and prognostic factors of the ADMYRE patients. HCT which included adult patients with r/r MM were extracted from Medidata Enterprise Data Store (MEDS). MEDS is a collection of thousands of previous clinical trials with patient-level data recorded through the Medidata electronic data capture system, Rave^®^.

### Objectives of the analysis

The objectives of this analysis were to assess the difference in OS by comparing the ADMYRE P + LD-DXM patients to the ECA POM + LD-DXM patients, and by comparing the ECA POM + LD-DXM patients to the ADMYRE LD-DXM alone patients. Safety, based on the occurrence of treatment-related adverse events (TRAEs), was also assessed. PFS, the primary endpoint in the ADMYRE study [3], was not included in this analysis because different International Myeloma Working Group criteria versions were used to calculate PFS events: without confirmation of disease progression (PD) in ADMYRE, and with confirmation of PD in the POM studies. Subsequent therapies could start without PD confirmation in the ADMYRE trial, while subsequent therapies could have been guided to start only after PD confirmation in the POM trials. Therefore, unbiased comparison for PFS based on the confirmation of PD between ADMYRE and POM trials cannot be fully achieved.

### Construction of the external control arms

Multiple steps for searching and selecting the qualified historical clinical studies to be used for ECA construction were implemented, including both trial-level and patient-level selection. The initial examination began with a search for all clinical trials of r/r MM where the study treatment contained POM + LD-DXM across major trial repositories (NIH/ClinicalTrials.gov, EudraCT, UMIN-CTR) and publication databases (PubMed) and required trial confirmation (via design description or publication of results) to enroll at least some patients treated with POM + LD-DXM; to recruit at least some patients between 1 June 2010 and 31 December 2017 (the ADMYRE trial was conducted between 29 June 2010 and 19 May 2017); and an interventional trial design. Eight contemporary trials (NCT01053949, NCT01311687, NCT01712789, NCT02654132, NCT02726581, NCT02916420, NCT02990338 and NCT03151811) met all the criteria described in Table 1 and were selected. Trial design elements (including objectives, endpoints measured, comparability of control arm treatments and masking status) were evaluated to ensure comparability with those of the ADMYRE trial.

**Table 1:** Key features of the ADMYRE trial and historical clinical trials

Study attributes	ADMYRE	Historical data ^a^
Trial phase	III	II/III
Intervention(s)	P + LD-DXM and LD-DXM alone	POM + LD-DXM
Intervention assignment	Parallel assignment	Single arm Parallel assignment
Masking	None (open label)	None (open label)
Study start year	2010	2009–2017
Study completion year	2017	2013–2022
Key endpoints	OS, PFS, ORR, safety	OS, PFS, ORR, safety
Planned follow-up duration (primary endpoints)	Approximately 5 years	Approximately 5 years
Region	Global	Global

^a^ Patients meeting the following requirements were included: patients studied in a trial covered by Medidata data sharing agreements; patients studied in a trial currently meeting Medidata completion eligibility requirements; patients studied in a trial that collects targeted endpoints and adequate planned follow-up, and patients who met the trial- and or patient-level project criteria.

Abbreviations: *LD-DXM* low-dose dexamethasone, *ORR* overall response rate, *OS* overall survival, *P* plitidepsin, *PFS* progression-free survival, *POM* pomalidomide

For all studies included in this analysis, patients provided written informed consent, and an independent ethics committee or institutional review board at each study center approved the study protocol. These studies were conducted in accordance with the Declaration of Helsinki and the International Conference on Harmonisation Guidelines for Good Clinical Practice.

After selecting the eight candidate HCTs, those studies that were covered by Medidata data sharing agreements and met Medidata completion eligibility requirements were further evaluated using the patient-level data. Only HCT patients who were assigned to receive the comparator of interest, POM + LD-DXM, and met similar key eligibility criteria from the ADMYRE study (Supplementary Table 1) were included in the potential ECA eligible pool. For ECA-eligible patients, POM was to be received orally at a dose of 4 mg on Day 1 through 21 and low-dose LD-DXM 40 mg orally (20 mg for patients > 75 years) per day on Day 1, 8, 15, and 22 q4wk. In ADMYRE trial, plitidepsin 5 mg/m² was administered as an intravenous infusion on Day 1 and 15 q4wk in combination with low-dose LD-DXM (40 mg orally on Day 1, 8, 15 and 22 q4wk vs. low-dose LD-DXM (40 mg orally on Day 1, 8, 15 and 22 q4wk).

Two separate ECAs were created using the propensity score method to conduct two sets of comparative analyses in matched populations:

- ECA1: P + LD-DXM (ADMYRE) was compared to the POM + LD-DXM arm.
- ECA2: POM + LD-DXM was compared to the LD-DXM arm (ADMYRE).

**Fig. 1:**
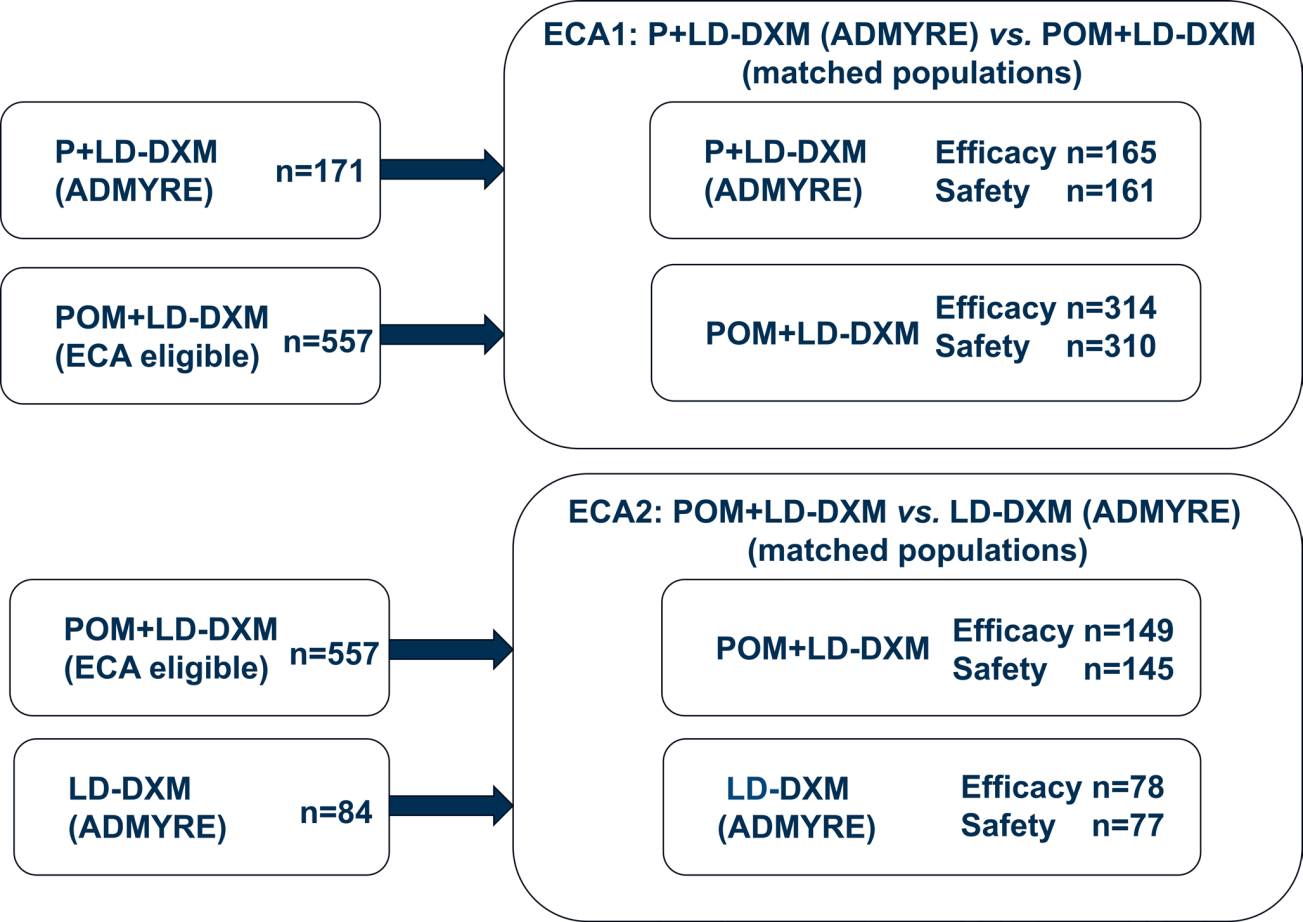
Design of comparative analysis using external control arms. Abbreviations: LD-DXM, low-dose dexamethasone; ECA, external control arm; P, plitidepsin; POM, pomalidomide

The resultant number of patients for these comparisons is summarized in Fig. 1. Median (range) patient age in the ADMYRE matched P + LD-DXM population was 64.0 (36.0–85.0) years and median (range) patient age in the POM + LD-DXM matched population (ECA1) was 64.9 (37.0–87.0) years. Median (range) patient age in the POM + LD-DXM matched population (ECA2) was 66.0 (37.0–87.0) years and median (range) patient age in the ADMYRE matched LD-DXM population was 66.0 (42.0–85.0) years (Supplementary Table 2).

### Propensity score and covariates assessment

Propensity scores, which is considered as a summary score combining all baseline characteristics, were defined as the probability of a patient belonging to the ADMYRE trial and being assigned to P + LD-DXM in ECA1 construction or LD-DXM in ECA2 construction, conditional on the baseline characteristics (i.e., potential confounders) using logistic regression, i.e., $\:p\left(x\right)=P\left(T=1\:|\:X=x\right)\:$where T = 1 if a patient was enrolled in ADMYRE and was assigned to P+LD-DXM (ECA1) or LD-DXM (ECA2), or T = 0 if a patient was assigned to receive POM+LD-DXM in HCTs; and X is a vector representing the covariates included in the propensity score model. Greedy nearest-neighbor matching algorithm [16] without replacement and a fixed 1-to-2 matching ratio was used. After a performance status was estimated for each patient from both ADMYRE and eligible ECA populations, the common support region was constructed and only subjects within this region were eligible for matching. The common support region was defined as the largest interval that contains propensity scores for subjects in both the ADMYRE and HCT groups, extended by 0.25 times the pooled estimate of the standard deviation of the logit of the propensity score in the ADMYRE and HCT groups, which is the default definition within the SAS PSMATCH procedure (SAS Institute Inc. 2017. SAS/STAT^®^ 14.3 User’s Guide. Cary, NC). Supplementary Fig. 1 presents two sets of box plots illustrating the distribution of propensity scores for patients that were included in the before- and after-matching sets. The comparability between the P + LD-DXM arm and POM+LD-DXM arm (ECA1) or between POM + LD-DXM arm (ECA2) and ADMYRE LD-DXM arm was improved after matching. The difference between the arms reduced substantially as evidenced by the placement of the median lines and mean markers.

The propensity score was used as a balancing score (see Supplementary Table 3 and Supplementary Table 4 for details on covariate selection). The distribution of measured baseline covariates was assessed in the after-matching analysis sets if was similar between the ADMYRE P + LD-DXM patients or ADMYRE LD-DXM randomized patients and the corresponding ECA patients in ECA1 and ECA2, respectively. Absolute standardized difference in covariate means was computed and compared following Austin’s notations [17]. The absolute standardized differences should generally be less than 0.25 [18]. An absolute standardized difference of less than 0.10 was taken to indicate a negligible difference in the mean or prevalence of a covariate between treatment groups [19]. The propensity score model that best achieved these thresholds was selected and implemented. After balance was achieved, the efficacy analysis of OS and safety analysis were performed in each ECA. Supplementary Fig. 2 shows the standardized differences after matching all fall within the highlighted range between − 0.10 and 0.10, indicating a negligible difference in the mean or prevalence of each covariate.

### Statistical methods

SAS software (version 9.4 or higher) was used for all statistical analyses. Summary descriptive statistics by treatment group are provided. For continuous variables, the descriptive statistics included the number of observations, mean, 95% confidence interval (CI), standard deviation, median, first quartile, third quartile, minimum, and maximum. For categorical variables, the descriptive statistics contained the frequency and percentage.

OS was defined as the time (in months) from the index date to death due to any cause or last contact date (censoring). The index date was defined as the date of randomization for patients from randomized studies, including ADMYRE and randomized HCT, or date of enrolment for patients from single-arm HCT. OS was estimated using the Kaplan-Meier method and compared between the ADMYRE and ECA patients using the after-matching analysis sets. Two-sided log-rank tests were used for testing the differences between the ADMYRE target groups and corresponding ECAs. The hazard ratio (HR) between the two treatment groups in each ECA analysis and its 95% CI using Wald’s confidence limits was estimated using a Cox regression model, including treatment group (i.e., ADMYRE vs. ECA) as a covariate. Ties were handled with Efron’s approximation. In ECA1 analysis, if the upper limit of the 95%CI for HR was below a prespecified non-inferiority margin of 1.3, it can be concluded that ADMYRE P + LD-DXM is non-inferior to POM + LD-DXM. This non-inferiority margin of 1.3 is based on a systematic review from 74 non-inferiority trials of cancer drug therapies, and on a systematic literature review of 285 published oncology clinical trials where the median non-inferiority margin was a HR of 1.29 [20, 21]. In the ECA2 analysis, if the upper limit of the 95%CI for HR was less than 1, POM + LD-DXM can be concluded as superior to LD-DXM (ADMYRE).

All statistical tests were two-sided at alpha level of 0.05.

## Results

### ECA1: P + LD-DXM arm (ADMYRE) *versus* POM + LD-DXM arm

**Fig. 2:**
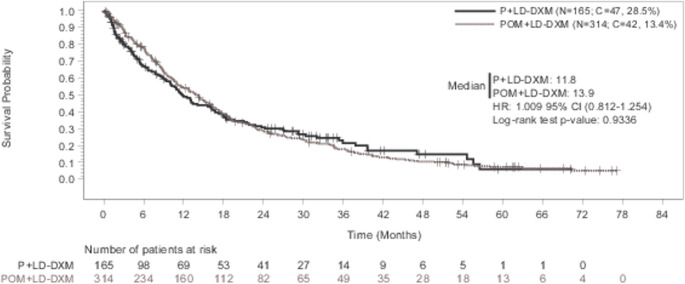
Kaplan-Meier curve of overall survival (ECA1 analysis): P + LD-DXM (ADMYRE) vs. POM + LD-DXM arm. Includes the patients from both ADMYRE P + LD-DXM arm and ECA eligible pool who are matched for the baseline covariates. ‘+’ indicates censoring. The hazard ratio (95%CI) is based on the Cox proportional hazards regression model with treatment group (P + LD-DXM vs. POM + LD-DXM) as a single covariate. Abbreviations: C, censored; CI, confidence interval; ECA, external control arm; HCT, historical clinical trial; HR, hazard ratio; KM, Kaplan-Meier; OS, overall survival; P + LD-DXM, plitidepsin plus low-dose dexamethasone; POM + LD-DXM, pomalidomide plus low-dose dexamethasone

The HR of OS for the P + LD-DXM arm (ADMYRE) relative to POM + LD-DXM (ECA1) was 1.009 (95%CI, 0.812–1.254) (two-sided log-rank test *p* = 0.9336) (Fig. 2). Kaplan-Meier plot shows that the P + LD-DXM arm and the POM + LD-DXM arm have similar survival estimates, with the curves close to one another and crossing at multiple points. As the 95%CI for the HR (0.812–1.254) falls below the pre-specified non-inferiority margin of 1.3, a strong signal of non-inferiority was found in OS for the ADMYRE P + LD-DXM treatment compared to POM + LD-DXM. Additionally, the median survival time and the log-rank p-value do not indicate signs of a difference between the arms.

A lower percentage of patients with any grade ≥ 3 TRAEs (50.3% vs. 63.9%) was found for the P + LD-DXM patients compared to the POM + LD-DXM patients (Table 2). Overall, the safety profile of P + LD-DXM (ADMYRE) when compared to POM + LD-DXM is characterized by a low rate of hematological TRAEs (e.g., grade ≥ 3 neutropenia 2.5% vs. 37.1%; grade ≥ 3 thrombocytopenia 2.5% vs. 13.2%) and infections (8.1% vs. 18.7%), while gastrointestinal, biochemical and musculoskeletal events were more common with P + LD-DXM.

**Table 2:** Treatment-related adverse events (≥ 5% of patients) (ECA1)

System Organ Class Preferred Term	All grades		Grade ≥ 3	
	*P* + LD-DXM (ADMYRE) (*n* = 161)	POM + LD-DXM (ECA1) (*n* = 310)	*P* + LD-DXM (ADMYRE) (*n* = 161)	POM + LD-DXM (ECA1) (*n* = 310)
Patients with any TRAE	138 (85.7)	265 (85.5)	-	-
Patients with any grade ≥ 3 TRAE	-	-	81 (50.3)	198 (63.9)
Blood and lymphatic system disorders	25 (15.5)	161 (51.9)	17 (10.6)	141 (45.5)
Anaemia	16 (9.9)	59 (19.0)	10 (6.2)	42 (13.5)
Neutropenia	4 (2.5)	127 (41.0)	4 (2.5)	115 (37.1)
Thrombocytopenia	4 (2.5)	57 (18.4)	4 (2.5)	41 (13.2)
Leukopenia	0	22 (7.1)	-	-
Gastrointestinal disorders	85 (52.8)	85 (27.4)	-	-
Nausea	61 (37.9)	22 (7.1)	-	-
Vomiting	28 (17.4)	5 (1.6)	-	-
Diarrhoea	23 (14.3)	25 (8.1)	-	-
Abdominal pain upper	10 (6.2)	4 (1.3)	-	-
Constipation	5 (3.1)	23 (7.4)	-	-
General disorders and administration site conditions	81 (50.3)	126 (40.6)	22 (13.7)	28 (9.0)
Fatigue	60 (37.3)	87 (28.1)	18 (11.2)	18 (5.8)
Oedema peripheral	18 (11.2)	28 (9.0)	-	-
Pyrexia	13 (8.1)	24 (7.7)	-	-
Infections and infestations	31 (19.3)	104 (33.5)	13 (8.1)	58 (18.7)
Pneumonia	7 (4.3)	33 (10.6)	4 (2.5)	29 (9.4)
Upper respiratory tract infection	3 (1.9)	16 (5.2)	-	-
Investigations	64 (39.8)	46 (14.8)	41 (25.5)	28 (9.0)
Blood CPK increased	26 (16.1)	0	23 (14.3)	0
ALT increased	25 (15.5)	2 (0.6)	14 (8.7)	1 (0.3)
AST increased	15 (9.3)	0	9 (5.6)	0
Electrocardiogram QT prolonged	9 (5.6)	0	-	-
Neutrophil count decreased	0	19 (6.1)	0	18 (5.8)
Metabolism and nutrition disorders	32 (19.9)	29 (9.4)	-	-
Decreased appetite	21 (13.0)	7 (2.3)	-	-
Hyperglycaemia	8 (5.0)	13 (4.2)	-	-
Musculoskeletal and connective tissue disorders	48 (29.8)	48 (15.5)	18 (11.2)	3 (1.0)
Myalgia	24 (14.9)	4 (1.3)	9 (5.6)	0
Muscular weakness	16 (9.9)	9 (2.9)	-	-
Muscle spasms	4 (2.5)	31 (10.0)	-	-
Nervous system disorders	16 (9.9)	62 (20.0)	-	-
Peripheral sensory neuropathy	1 (0.6)	19 (6.1)	-	-
Tremor	0	18 (5.8)	-	-
Psychiatric disorders	20 (12.4)	55 (17.7)	-	-
Insomnia	11 (6.8)	21 (6.8)	-	-
Respiratory, thoracic and mediastinal disorders	22 (13.7)	67 (21.6)	-	-
Dyspnoea	11 (6.8)	24 (7.7)	-	-
Cough	4 (2.5)	18 (5.8)	-	-
Skin and subcutaneous tissue disorders	8 (5.0)	49 (15.8)	-	-
Rash	3 (1.9)	16 (5.2)	-	-

Data shown are n (%) of patients. Ordered by frequency in the first column

A dash (“-“) indicates that the value did not reach the threshold of 5% of patients

TRAEs were coded using MedDRA version 16.0. For a patient who had multiple occurrences of the same SOC and PT, the patient was counted only once for the corresponding SOC and PT. SOC is presented alphabetically and PTs within each SOC are presented in descending order of prevalence in the P + LD-DXM arm

Abbreviations: *ALT* alanine aminotransferase, *AST* aspartate aminotransferase, *CPK* creatine phosphokinase, *ECA* external control arm, *MedDRA* Medical Dictionary for Regulatory Activities, *P + LD-DXM* plitidepsin plus low-dose dexamethasone, *POM + LD-DXM* pomalidomide plus low-dose dexamethasone, *PT* preferred term, *SOC* system organ class, *TRAE* treatment-related adverse event

### ECA2: POM + LD-DXM *versus* LD-DXM Arm (ADMYRE)

**Fig. 3:**
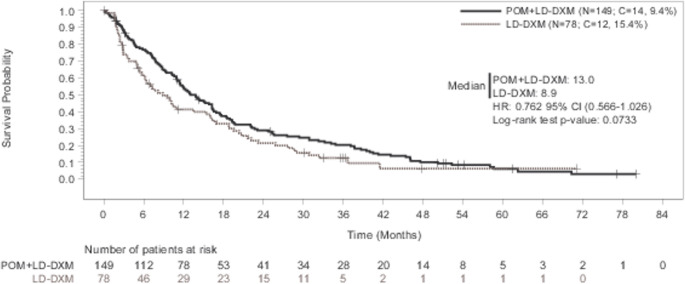
Kaplan-Meier curve of overall survival (ECA2 analysis): POM + LD-DXM vs. LD-DXM (ADMYRE). Includes the patients from both ADMYRE LD-DXM arm and ECA eligible pool who are matched for the baseline covariates. The hazard ratio (95%CI) is based on the Cox proportional hazards regression model with treatment group (POM + LD-DXM vs. LD-DXM) as a single covariate. Abbreviations: C, censored; CI, confidence interval; LD-DXM, low-dose dexamethasone; ECA, external control arm; HCT, historical clinical trial; HR, hazard ratio; KM, Kaplan-Meier; OS, overall survival; POM, pomalidomide

The HR of OS for the POM + LD-DXM arm (ECA2) relative to the ADMYRE LD-DXM arm was 0.762 (95%CI, 0.566–1.026) (two-sided log-rank test *p* = 0.0733) (Fig. 3).

POM + LD-DXM was associated with a higher rate of grade ≥ 3 TRAEs compared to the control arm used in the ADMYRE (LD-DXM): 60.0% vs. 10.4% of patients (Table 3), particularly notable for hematological and infection events.

**Table 3:** Treatment-related adverse events (≥ 5% of patients) (ECA2)

System Organ Class Preferred Term	All grades		Grade ≥ 3	
	POM + LD-DXM (ECA2) (*n* = 145)	LD-DXM (ADMYRE) (*n* = 77)	POM + LD-DXM (ECA2) (*n* = 145)	LD-DXM (ADMYRE) (*n* = 77)
Patients with any TRAE	124 (85.5)	35 (45.5)	-	-
Patients with any grade ≥ 3 TRAE	-	-	87 (60.0)	8 (10.4)
Blood and lymphatic system disorders	72 (49.7)	1 (1.3)	64 (44.1)	1 (1.3)
Neutropenia	55 (37.9)	0	49 (33.8)	0
Anaemia	34 (23.4)	1 (1.3)	25 (17.2)	1 (1.3)
Thrombocytopenia	25 (17.2)	0	16 (11.0)	0
Leukopenia	8 (5.5)	0	-	-
Gastrointestinal disorders	42 (29.0)	13 (16.9)	-	-
Constipation	15 (10.3)	1 (1.3)	-	-
Diarrhoea	13 (9.0)	2 (2.6)	-	-
Nausea	8 (5.5)	7 (9.1)	-	-
Dyspepsia	4 (2.8)	4 (5.2)	-	-
General disorders and administration site conditions	59 (40.7)	10 (13.0)	14 (9.7)	1 (1.3)
Fatigue	40 (27.6)	7 (9.1)	-	-
Pyrexia	14 (9.7)	2 (2.6)	-	-
Oedema peripheral	12 (8.3)	1 (1.3)	-	-
Infections and infestations	46 (31.7)	8 (10.4)	29 (20.0)	3 (3.9)
Pneumonia	19 (13.1)	0	17 (11.7)	0
Bronchitis	8 (5.5)	0	-	-
Investigations	27 (18.6)	0	18 (12.4)	0
Neutrophil count decreased	14 (9.7)	0	14 (9.7)	0
Musculoskeletal and connective tissue disorders	25 (17.2)	9 (11.7)	-	-
Muscle spasms	18 (12.4)	1 (1.3)	-	-
Nervous system disorders	29 (20.0)	5 (6.5)	-	-
Peripheral sensory neuropathy	10 (6.9)	0	-	-
Psychiatric disorders	26 (17.9)	11 (14.3)	-	-
Insomnia	10 (6.9)	8 (10.4)	-	-
Respiratory, thoracic and mediastinal disorders	29 (20.0)	5 (6.5)	-	-
Dyspnoea	12 (8.3)	2 (2.6)	-	-
Cough	8 (5.5)	1 (1.3)	-	-
Skin and subcutaneous tissue disorders	27 (18.6)	5 (6.5)	-	-
Rash	8 (5.5)	0	-	-

Data shown are n (%) of patients. Ordered by frequency in the first column

A dash (“-“) indicates that the value did not reach the threshold of 5% of patients

TRAEs were coded using MedDRA version 16.0. For a patient who had multiple occurrences of the same SOC and PT, the patient was counted only once for the corresponding SOC and PT. SOC is presented alphabetically and PTs within each SOC are presented in descending order of prevalence in the P + LD-DXM arm

Abbreviations: *ALT* alanine aminotransferase, *AST* aspartate aminotransferase, *CPK* creatine phosphokinase, *ECA* external control arm, *MedDRA* Medical Dictionary for Regulatory Activities, *P + LD-DXM* plitidepsin plus low-dose dexamethasone, *POM + LD-DXM* pomalidomide plus low-dose dexamethasone, *PT* preferred term, *SOC* system organ class, *TRAE* treatment-related adverse event

## Discussion

Overall survival results showed non-inferiority for patients assigned to be treated with P + LD-DXM from ADMYRE [3] compared to matched patients treated with POM + LD-DXM in HCT. No difference in OS was observed between P + LD-DXM and POM + LD-DXM (HR = 1.009; 95%CI, 0.812–1.254). Additionally, the log-rank test p-value of 0.9336 does not indicate a statistically significant difference in OS.

The treatment effect for POM + LD-DXM obtained by comparing POM + LD-DXM in HCT to the LD-DXM control data from the ADMYRE trial (HR = 0.762; 95%CI, 0.566–1.026) was similar to the treatment effect previously demonstrated for P + LD-DXM compared to LD-DXM in the ADMYRE trial (HR = 0.797, 95%CI, 0.596–1.067) [3].

Subsequent line therapies may have an impact on overall survival outcomes. The fact that trials were contemporary might help to reduce the diversity of further therapies and then its likely effect on survival. Nevertheless, it has to be remarked that the similar effect on OS observed for the arms evaluated was obtained despite a lower percentage of patients treated in the ADMYRE trial arms received subsequent therapy: 47.9% (P + LD-DXM) vs. 65.6% (POM + LD-DXM) in ECA1; and 55.1% (POM + LD-DXM) vs. 71.1% (LD-DXM) in ECA2 (Supplementary Table 2).

In the ECA1 patient-level comparison, the safety profile of P + LD-DXM (ADMYRE) when compared to POM + LD-DXM (ECA1) is characterized by a low rate of hematological adverse events and infections, with a lower percentage of grade ≥ 3 TRAEs, while gastrointestinal, biochemical and musculoskeletal events were more common with P + LD-DXM. The different safety profile of plitidepsin to that of other agents approved for r/r MM like pomalidomide should be noted. Plitidepsin is not associated with renal impairment, neutropenia, thrombocytopenia, thromboembolism, rash or neurotoxicity, or infections, as is the case with many agents used in r/r MM in clinical trials contemporaneous to ADMYRE [15, 22–27]. This differentiated safety profile makes plitidepsin a valuable treatment option in patients known to be at risk of particular adverse event known to occur with the use of these agents.

eEF1A2, a protein which is overexpressed in MM, has been identified as the primary target for plitidepsin [2, 28]. Of note, all recently introduced new anti-MM drugs have mechanisms of activity not targeting eEF1A2.

In the other patient-level comparison (ECA2), POM + LD-DXM was associated with a higher rate of events compared to the control arm used in ADMYRE (LD-DXM). Therefore, the finding of more toxicity reported for P + LD-DXM in the ADMYRE trial [3], which was expected from a combination when compared to a monotherapy, was also observed with POM + LD-DXM.

## Conclusions

This patient-level external data comparison shows that P + LD-DXM is non-inferior to POM + LD-DXM in terms of overall survival in a similar population than that evaluated in the AMDYRE trial. The safety profile of P + LD-DXM is characterized by a low rate of hematological adverse events and infections compared to POM + LD-DXM. A new therapy with a novel mechanism of action that shows a PFS benefit, as proven in ADMYRE study [3], along with a likelihood of survival benefit in a largely treatment-refractory population, provide plitidepsin with a specific place into the available options of the haemato-oncologist treating r/r MM and could be considered a new therapeutic option in this setting.

## Supplementary Information

Below is the link to the electronic supplementary material.

Supplementary Material 1 (DOCX 168 KB)

## Data Availability

The original contributions presented in the analysis have been included in the article; further inquiries can be directly addressed to the corresponding author.
