# Supplementary material for: Plitidepsin in combination with dexamethasone (ADMYRE trial) versus an external control arm of pomalidomide plus dexamethasone in patients with relapsed/refractory multiple myeloma
Source: Ann Hematol. 2026 Jan 17;105(1):26. doi: 10.1007/s00277-026-06811-w (PMC12811270; doi:10.1007/s00277-026-06811-w)
Supplement: Supplementary file 1 — Supplementary Material 1 (DOCX 168 KB) [file 277_2026_6811_MOESM1_ESM.docx]

**SUPPLEMENTARY INFORMATION**

Supplementary Table 1. Key eligibility criteria of historical control patients at study baseline.

| **Criterion** |
| --- |
| Age ≥ 18 years, ECOG performance status ≤ 2 |
| Patients must have relapsed or refractory MM after ≥ 3 and ≤ 6 prior therapeutic lines of therapies for MM |
| Prior treatment with bortezomib-containing regimens and lenalidomide-containing or thalidomide-containing regimens |
| Patients must have measurable disease according to IMWG |
| No major procedures and anti-myeloma treatments or other therapy that is or may be active against MM at least two-week prior to study entry |
| Enrolment dates between 1 June 2010 and 31 December 2017 |
| Exclude: Concomitant diseases/conditions that are deemed unsuitable to be enrolled in the study by investigators |
| Exclude: Clinical evidence and/or lab data (corrected serum calcium > 11.5 mg/dL) of hypercalcemia prior to or on baseline |
| Exclude: Prior receipt of daratumumab |
| Exclude: Biclonal MM |
| Abbreviations: ECOG, Eastern Cooperative Oncology Group; IMWG, International Myeloma Working Group; MM, multiple myeloma. |

Supplementary Table 2. Age and number of patients receiving subsequent therapy in the matched populations.

|  | **P+LD-DXM**  **(ADMYRE)**  **(n=165)** | **ECA1**  **(n=314)** | **ECA2**  **(n=149)** | **LD-DXM**  **(ADMYRE)**  **n=78)** |
| --- | --- | --- | --- | --- |
| **Age (years)** | | | | |
| **Median** | 64.0 | 64.9 | 66.0 | 66.0 |
| **Min – Max** | 36.0 – 85.0 | 37.0 – 87.0 | 37.0 – 87.0 | 42.0 – 85.0 |
| **Received subsequent therapy (n and % of patients)** | | | | |
| **Yes** | 79 (47.9) | 206 (65.6) | 106 (71.1) | 43 (55.1) |
| **No** | 86 (52.1) | 108 (34.4) | 43 (28.9) | 35 (44.9) |
| LD-DXM, low-dose dexamethasone; Min, minimum; Max, maximum; P, plitidepsin. | | | | |

Supplementary Table 3. Clinically important baseline covariates included in the propensity score model.

| **Baseline variable ^a^** | **Type of collected variable (values/unit)** |
| --- | --- |
| **Age** | Categorical (< 75 years, ≥ 75 years) |
| **Gender** | Categorical (female, male) |
| **Body surface area (BSA)** | Continuous (m^2^) |
| **ISS Stage ^b^** | Categorical (I, II/Unknown, III) |
| **MM type** | Categorical (IgA, IgG, Other) |
| **ECOG performance** | Categorical (0, ≥ 1) |
| **Plasma cells** | Continuous (%) |
| **Bone lytic lesions** | Categorical (present, absent) |
| **Plasmacytomas** | Categorical (present, absent) |
| **Time from diagnosis to index date ^c^** | Continuous (months) |
| **Time from last progression to index date ^c^** | Continuous (months) |
| **Corrected serum calcium** | Continuous (mg/dL) |
| **LDH ^b^** | Categorical (≤ 1 x ULN/unknown ^d^, > 1 x ULN ^d^) |
| **Creatinine** | Continuous (mg/dL) |
| **Number of prior lines of anticancer treatment** | Categorical (≤ 4, > 4) |
| **Previous stem cell transplant** | Categorical (Any, None) |
| **Refractory status to last prior therapy ^e^** | Categorical (Refractory/unknown, relapsed/unknown ^f^) |
| **Refractory status to prior bortezomib ^b^** | Categorical (refractory, relapsed/unknown ^f^) |
| **Refractory status to prior thalidomide/lenalidomide ^2,3^** | Categorical (refractory, relapsed/unknown ^f^) |
| **Refractory status to prior PIs ^b^** | Categorical (refractory, relapsed/unknown ^f^) |
| ^a^ Baseline was defined as the last non-missing value prior to or on the treatment start date or the last non-missing value collected during screening/enrollment visits if treatment start date was not available.  ^b^ The level unknown was grouped with was the most prevalent level of the variable.  ^c^ Index date was defined as the date of randomization for patients from randomized studies, including ADMYRE and randomized HCTs, or date of enrollment for patients from single-arm HCTs.  ^d^ ULN, when not available, was imputed as 250 IU/L which was approximately the median ULN used across plitidepsin studies.  ^e^ In ECA1, unknown was grouped with the “relapsed” category due to it being the most prevalent level in both arms; in ECA2, unknown was grouped with the “refractory2 category in the LD-DXM arm and combined with the “relapsed” category in the ECA eligible arm, the category representing the larger proportion of patients within each treatment arm, respectively.  ^f^ Relapsed contains the following categories: relapsed, relapsed and refractory.  Abbreviations: BSA, body surface area; ECOG, Eastern Cooperative Oncology Group; Ig, immunoglobulin; ISS, International Staging System; LD-DXM, low-dose dexamethasone; LDH, lactate dehydrogenase; MM, multiple myeloma; PI, proteasome inhibitor; ULN, upper limit of normal. | |

Supplementary Table 4. Baseline covariates considered but excluded from the propensity score model.

| **Baseline variable** | **Reason for exclusion** |
| --- | --- |
| **Race** | Data was not consistently collected in target trials. |
| **Ethnicity** | Data was not consistently collected in target trials. |
| **Number of lesions** | Bone lytic lesions (present, absent) and plasmacytomas (present, absent) were collected and included in the propensity score model. Number of lesions provided limited additional information. |
| **Sum of the dimensions of plasmacytomas** | Plasmacytomas (present, absent) was collected and included in the propensity score model, but the number of patients with plasmacytomas was limited in target trials and HCTs and the sum of dimensions was not consistently recorded. |
| **MM type at diagnosis** | Not collected in target trials or HCTs. Instead, MM type at baseline was collected and included in the propensity score model. |
| **Durie-Salmon stage** | Data was not consistently collected in HCTs and could be difficult to derive using laboratory values. ISS stage was collected or derived and included in the propensity score model. |
| **Refractory status to prior IMiD therapy** | IMiD includes only pomalidomide, lenalidomide, and thalidomide. Since HCTs did not include prior pomalidomide, this variable was a duplicate of the refractory status to lenalidomide/thalidomide which was already included in Supplementary Table 2. |
| **Cytogenetic risk** | Data was not consistently collected in target trials or HCTs. |
| **Hemoglobin** | In ADMYRE, baseline hemoglobin may have been recorded pre- or post-transfusion for different patients; therefore, the data collected was not a reliable representation of a patient's true value. Additionally, hemoglobin was expected to have limited prognostic value. |
| Abbreviations: HCT, historical clinical trials; IMiD, immunomodulatory drugs; MM, multiple myeloma. | |

Supplementary Fig. 1. Box Plot of Propensity Score Distribution by Treatment Group.

1. Before and after matching (ECA1)


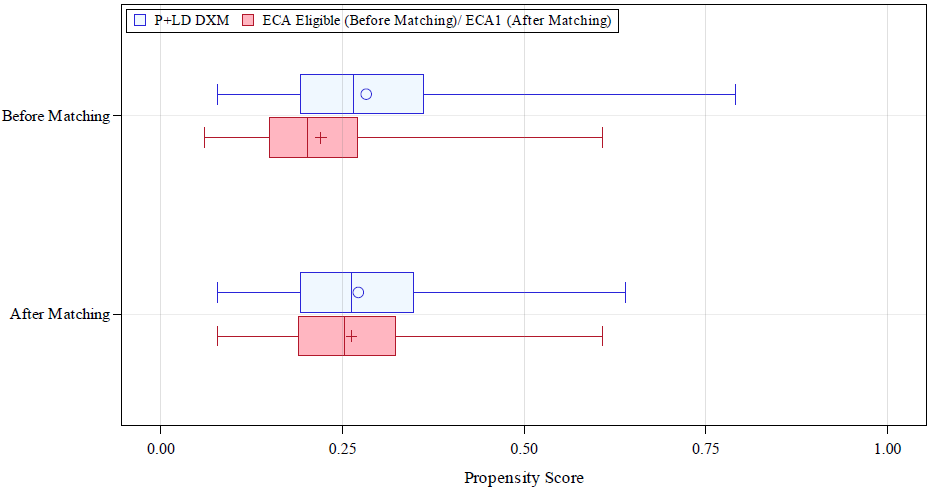


Abbreviations: ECA, external control arm, P+LD DXM, plitidepsin plus low-dose dexamethasone. Within the boxes, the circle and '+' symbols represent the mean and the vertical lines the median.

1. Before and after matching (ECA2)


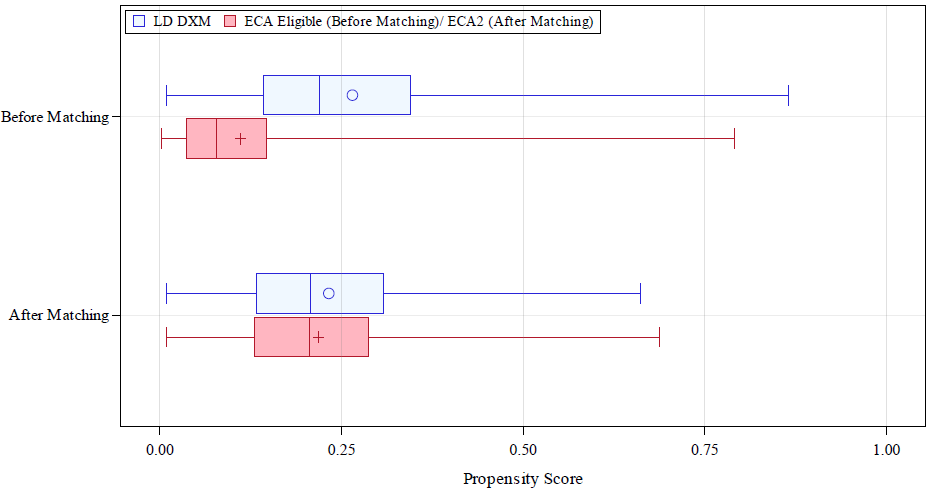


Abbreviations: ECA, external control arm; LD-DXM, low-dose dexamethasone. Within the boxes, the circle and '+' symbols represent the mean and the vertical lines the median.

Supplementary Fig. 2. Standardized differences in baseline demographics and disease characteristics between treatment groups.

1. Before and after matching (ECA1)


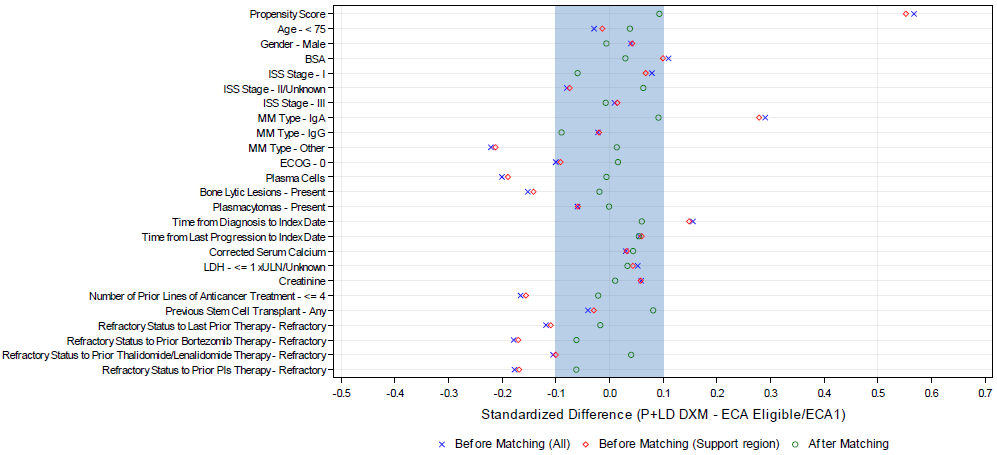


Abbreviations: BSA, body surface area, ECA, external control arm, ECOG, Eastern Cooperative Oncology Group, Ig, immunoglobulin, ISS, International Staging System, LDH, lactate dehydrogenase, MM, multiple myeloma, P+LD DXM, plitidepsin plus low-dose dexamethasone; PI, proteasome inhibitor, ULN, upper limit of normal.

1. Before and after matching (ECA2)


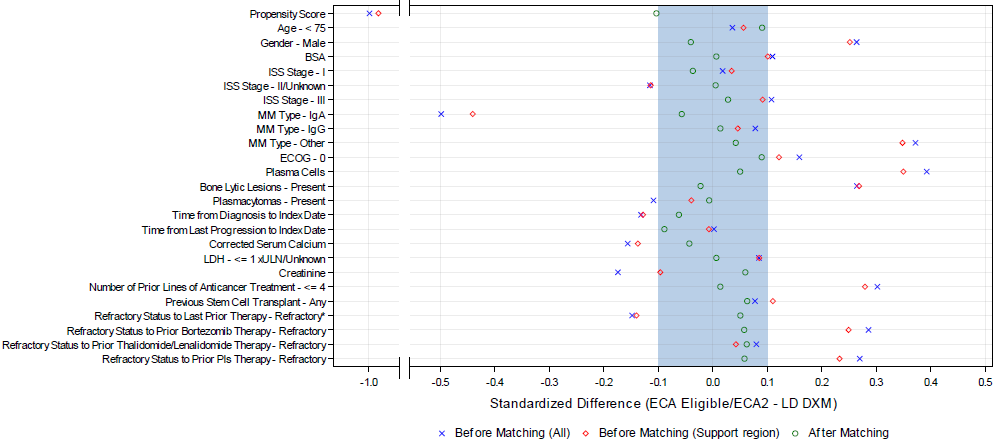


* Unknown refractory status was combined with the refractory category in the LD DXM arm and combined with the relapsed category in the ECA Eligible arm, the category representing the larger proportion of patients within each treatment arm, respectively.

Abbreviations: BSA, body surface area, ECA, external control arm, ECOG, Eastern Cooperative Oncology Group, Ig, immunoglobulin, ISS, International Staging System, LDH, lactate dehydrogenase, MM, multiple myeloma, P+LD DXM, plitidepsin plus low-dose dexamethasone; PI, proteasome inhibitor, ULN, upper limit of normal.
